
JOURNAL INFORMATION
==============================
NLM Title Abbreviation: ScientificWorldJournal
Journal ID: ScientificWorldJournal
NLM Journal ID: 101131163
Journal ID: TSWJ

The Scientific World Journal

ISSN: 2356-6140
EISSN: 1537-744X
Publisher: Wiley

ARTICLE INFORMATION
==============================
PMCID: PMC5901277
PMID: 17982572
DOI: 10.1100/tsw.2007.226
Article ID: 395095
Article version: 1
Subjects: Editorial

Evaluating the Functional Importance of Neuroadaptions in Addiction

Volman Susan F. *
National Institute on Drug Abuse, National Institutes of Health, Department of Health and Human Services, Bethesda, MD 20892, USA
*Susan F. Volman: svolman@nida.nih.govAcademic Editors: D. Shurtleff and S. Ferre

Publication date: 2007
Electronic publication date: 2007 Nov 2
Volume: 7
First page: 4
Last page: 8
Received 2007 Aug 2; Accepted 2007 Aug 2
Copyright: Copyright © 2007 Susan F. Volman.
Copyright year: 2007
License: This is an open access article distributed under the Creative Commons Attribution License, which permits unrestricted use, distribution, and reproduction in any medium, provided the original work is properly cited.
License URL: https://creativecommons.org/licenses/by/3.0/

Funding: D.C. Department of Mental Health

==============================
Editorial
NIDA Special Issue on Frontiers in Addiction Research
*TheScientificWorldJOURNAL* (2007) 7(S2), 4–8
ISSN 1537-744X; DOI 10.1100/tsw.2007.226

TheScientificWorldJOURNAL
www.thescientificworld.com

### Evaluating the Functional Importance of Neuroadaptions in Addiction

Susan F. Volman

*National Institute on Drug Abuse, National Institutes of Health, Department of Health and Human Services, Bethesda, MD 20892*

**E-mail**: svolman@nida.nih.gov

*Received August 2, 2007; Accepted August 2, 2007; Published November 2, 2007*

---

Studies in animal models, and to a lesser extent in humans, have revealed a wide range of long-lasting adaptations in neuronal excitability, synaptic function, neuron architecture, and expression of genes associated with neural plasticity after exposure to drugs or alcohol. Similar alterations have been observed in individuals with differential genetic vulnerabilities for substance abuse. As the techniques for identifying neural adaptations have multiplied and data have accumulated at a rapid pace, it has become clear that there is a formidable problem in determining which adaptations are critical for establishing and maintaining addiction, and which are not[1,2]. Two NIDA-supported symposia at the 2007 Society for Neurosciences (SFN) meeting address this problem. A symposium in the National Institute on Drug Abuse (NIDA) Frontiers in Addiction Research Miniconvention, “Neuronal Adaptations and Counteradaptations”, showcases three very different approaches in presentations by Robert Malenka, L. Judson Chandler, and Lorna Role. An SFN symposium, “Reconciling Molecular and Electrophysiological Evidence of Cocaine-Induced Neural Plasticity”, focuses on the apparent contradiction between molecular and neurophysiological changes in nucleus accumbens and recent studies that attempt to resolve this contradiction in presentations by Marina Wolf, Mark Thomas, Xiu-Ti Hu, and Laura Peoples. These symposia are represented by two papers in this issue[3,4].

### NEURONAL ADAPTATIONS AND COUNTERADAPTATIONS

Homeostatic plasticity has received recent attention in attempts to identify which neuroadaptations are critical for establishing and maintaining addiction. Homeostatic mechanisms are particularly important to consider when evaluating molecular changes observed after exposure to abused substances. All these substances mimic, enhance, or interact in other ways with the effects of endogenous neurotransmitters, and it is well known that, in the normal brain, self-regulatory mechanisms exist to maintain the levels of neurotransmitters, receptors, and neuronal excitability within a normal operating range. Therefore, it is reasonable to expect that some cellular changes observed after drug exposure represent such regulatory mechanisms. A recent study by Malenka and collaborators suggests that activation of the cyclic AMP response element binding protein (CREB) in the nucleus accumbens (NAc) by cocaine is, functionally, a homeostatic “counteradaptation” that opposes cocaine-induced decreases in the excitability of medium spiny neurons (MSNs) in the NAc[5]. The investigators used recombinant viral vectors to express constitutively active or dominant-negative forms of CREB in MSNs. Expression of active CREB increased the excitability of these neurons, whereas expression of the dominant-negative form had the opposite effect. In rats that had been exposed to repeated cocaine injections, the excitability of MSNs was decreased, but this effect was reversed when the expression of active CREB was induced after cocaine

©2007 with author.
Published by TheScientificWorld; www.thescientificworld.com treatment. The study further showed that decreasing MSN excitability alone, by overexpression of an exogenous potassium channel, was sufficient to mimic cocaine behavioral sensitization. Taken together, the results indicate that CREB activation in the NAc helps to limit the sensitized response to repeated cocaine exposure produced, at least in part, by cocaine-induced decreases in MSN excitability. Findings such as these illustrate the complexity of identifying which cellular changes in which brain areas mediate addiction and which are secondary events, especially for ubiquitous transcription factors such as CREB[6].

Structural modifications of dendrites and dendritic spines have long been associated with experience-dependent changes in behavior. However, there have been relatively few investigations of changes in neuronal architecture in relation to the development of addiction to drugs or alcohol, despite the fact that addiction is a profound example of an experience-dependent change in behavior and psychological function[7]. Chandler and colleagues have recently begun to investigate structural plasticity and its molecular basis in response to alcohol exposure[3,8,9]. Their observations in hippocampal neurons support a model in which chronic ethanol exposure induces homeostatic increases of NMDA receptors and other molecular components of the postsynaptic density as an adaptive response to reductions in synaptic signaling produced by ethanol. Further, their results suggest that these changes in protein localization promote the enlargement of dendritic spines. Chandler's evidence for spine enlargement in the hippocampus after exposure to alcohol, along with extensive evidence for structural plasticity in dendrites and dendritic spines induced by psychostimulants and nicotine from studies by Robinson and Kolb[7], reflect the profound ability of substances of abuse to alter neuronal connectivity.

Another approach for discovering neural mechanisms of drug dependence has been to identify genetic differences among individuals with differential vulnerability for addiction. One interesting example of this approach stems from the observation of the excessive prevalence of smoking among schizophrenic patients. This association has led to a search for evidence that alterations in nicotinic receptors or their expression are involved in the pathophysiology of schizophrenia and nicotine dependence in schizophrenic patients[10,11]. Role's research on the development of nicotinic synapses led to the discovery of neuregulin 1 (Nrg1) as a key regulator of the α7 nicotinic acetylcholine receptor (α7-nAChR). Genetic variations in the α7-nAChR have been linked with sensory gating deficits associated with schizophrenia, and recently, genetic variants of Nrg1 have been associated with heritable forms of schizophrenia. Role suggests that α7 and Nrg1 variants may predispose to nicotine dependence and that convergent effects of Nrg1 and a7 expression underlie the comorbidity of nicotine abuse and susceptibility to neuropsychiatric disorders such as schizophrenia.

### RECONCILING MOLECULAR AND ELECTROPHYSIOLOGICAL EVIDENCE OF COCAINE-INDUCED NEURAL PLASTICITY

The hallmark symptoms of drug dependence include a compulsive desire to seek and take drugs, reduced motivation to respond to natural rewards, and the long-lasting, often lifetime, susceptibility to relapse in response to drug-related environmental cues, stress, and other triggers. Many lines of behavioral and neurobiological research have suggested that neural adaptations in frontal-striatal circuits are a key mechanism in establishing and maintaining drug dependence[12,13]. However, until recently, it has been difficult to reconcile the sometimes contradictory data to establish a model of how specific cellular neuroplasticity relates to behavioral outcome. A recent review by Kalivas and Hu[14] and the symposium at the 2007 SFN Annual Meeting combine new experimental evidence and ideas to resolve these apparent contradictions, and explain how psychostimulant-induced neuroplasticity in the NAc contributes to addiction.

The contradictory evidence can be seen in the results from electrophysiological studies, which show that repeated exposure to psychostimulants reduces the activity of NAc neurons[4,14], whereas other evidence suggests that drug exposure enhances excitation of NAc neurons, particularly in response to inputs from the prefrontal cortex (PFC). Such evidence includes an increased cell-surface expression of

AMPA glutamate receptors on MSNs in the NAc, increased glutamate release from PFC terminals, increased numbers of dendritic spines after drug exposure, and increased neuronal firing in awake, behaving animals during drug self-administration and to drug-related cues in withdrawal.

To explain why the consequences of repeated cocaine can be either excitatory or inhibitory one may propose that the results of specific experiments depend on drug history, whether drug is self or experimenter administered, the duration of abstinence, or the area of NAc being examined. Recent studies by the Thomas and Wolf laboratories provide excellent examples of how drug history can influence synaptic plasticity[15,16,17]. Thomas and colleagues recorded whole-cell synaptic physiology in NAc shell neurons in mouse brain slices and demonstrated a progression of synaptic potentiation and depression in glutamatergic synaptic strength after repeated *in vivo* exposure to cocaine. In their previous studies, they had observed synaptic depression in NAc after repeated cocaine exposure and a period of abstinence, but the animals had all been tested for sensitization with a drug challenge shortly before brain slices were prepared for recording. In the new study, the investigators specifically tested whether the cocaine challenge affected the plasticity of NAc excitatory synapses. They found that repeated drug exposure followed by a 10-14 day drug-free period produced a robust potentiation of AMPAR-mediated synaptic transmission. However, a single re-exposure to cocaine triggered synaptic depression, abruptly reversing the initial potentiation. This latter finding demonstrates that a past history of repeated drug exposure can dramatically alter the type of synaptic plasticity elicited by a single drug dose.

Wolf and colleagues generated parallel biochemical findings using a protein cross-linking assay to distinguish between cell surface and intracellular AMPA receptors. NAc tissue taken from cocaine-sensitized animals after 14 of 21 days of abstinence had an increased surface/intracellular ratio of AMPAR subunits. No increases were seen in tissue from animals that failed to sensitize to the same treatment regimen, nor in tissue tested after only 1 day of withdrawal. However, in NAc tissue from sensitized rats that were re-exposed to cocaine after 14 days of withdrawal and killed 24 hours later, the AMPA receptor surface/intracellular ratio was reduced below control levels. These results indicate that AMPA receptors redistribute to the cell surface during cocaine withdrawal but internalize within 24 hours of cocaine challenge[16,17].

A study by Antonello Bonci and his colleagues illustrates that the nature of drug-related synaptic plasticity may depend on the area examined in the NAc[18]. In this study, rats were trained to self-administer cocaine, and recordings were made from both NAc core and shell. One day after drug exposure, they found that long-term depression (LTD) was inhibited in both the core and shell regions, but after 21 days of abstinence, LTD remained inhibited only in the NAc core.

Another approach to reconcile contradictory findings regarding increased AMPA receptor expression and neuronal hypoactivity takes homeostatic synaptic scaling into account. Studies by Hu and his colleagues in recordings from dissociated NAc neurons and brain slices, conducted in the laboratory of the late Francis White, have consistently shown that ionic currents mediated by various $K^+$, $Na^+$, and $Ca^{++}$ channels, are altered by repeated cocaine exposure to decrease the excitability of MSNs in early withdrawal[19]. Wolf and colleagues propose that this decrease in excitability, coupled with hypoactivity in cortical areas sending excitatory projections to the NAc, leads to a compensatory increase in postsynaptic AMPA receptors via synaptic scaling, a form of homeosotatic plasticity[16]. This mechanism would explain electrophysiological and biochemical evidence indicating increased AMPA receptor transmission in the NAc after cocaine withdrawal as described above.

Finally, the contradictory findings might be reconciled by taking into account the fact that, behaviorally, drug exposure increases responses to drug-related cues and drug seeking while at the same time it decreases the value of nondrug reward. Peoples has proposed a “differential inhibition hypothesis”, which posits that specific adaptations that facilitate responsiveness to excitatory afferents combine with decreased intrinsic excitability to enhance signals that promote drug seeking and weaken signals that normally promote other motivated behaviors[4,20]. The hypothesis, which is described in detail in this issue, combines evidence from the sorts of cellular level studies described above with results from single neuron recording experiments in awake animals in a number of self-administration paradigms that have been conducted in her laboratory and by others[21,22,23,24].

### FUTURE DIRECTIONS, GAPS AND OPPORTUNITIES

Long-lasting alterations in neuron excitability and synaptic function have a profound effect on behavior and information processing in the brain, but understanding the causes and consequences of these adaptations is particularly complex in relation to substance abuse. Neural adaptations observed after exposure to drugs or alcohol may be direct responses to the pharmacological effects of the drug, indirect effects of engaging brain systems involved in reward processing and learning, or compensatory, homeostatic responses to changes in excitability caused by drug exposure. Drugs of abuse or alcohol can also interfere with ongoing processes of neural adaptation, such as activity-dependent plasticity involved in learning or development.

The approaches described above have been fruitful in identifying cellular changes that mediate addiction. NIDA encourages more research along these lines, particularly with approaches that combine cellular level studies, neurophysiology, and behavioral analysis. To test the functional significance of neuroadaptations produced by drug exposure, more studies could take advantage of relapse/reinstatement models and comparisons between contingent and non-contingent drug administration. Another promising avenue is the use of computational models, particularly those that can test the sensitivity of the neural circuits involved in addiction to the types of cellular adaptations observed in experimental studies and provide testable hypotheses for further experimentation [e.g.25,26,27,28,29,30]. Recent initiatives at NIDA have also emphasized the importance of gene/environment/developmental interactions, social neuroscience, and epigentic processes, for understanding the neural basis of addiction and discovering targets for treatment.

### REFERENCES

1. Kalivas, P.W. (2005) How do we determine which drug-induced neuroplastic changes are important? *Nat. Neurosci*. **8,** 1440–1401.
2. Hyman, S.E., Malenka, R.C., and Nestler, E.J. (2006) Neural mechanisms of addiction: the role of reward-related learning and memory. *Annu. Rev. Neurosci*. **29**, 565–598.
3. Chandler, L.J. and Mulholland, J. (2007) The Thorny Side of Addiction: Adaptive Plasticity and Dendritic Spines. *TheScientificWorldJOURNAL*. **7(S2)**, 9-21.
4. Peoples, L.L, Kravitz, A.V., and Guillem, K. (2007) The Role of Accumbal Hypoactivity in Cocaine Addiction. *TheScientificWorldJOURNAL*. **7(S2)**, 22-45.
5. Dong, Y., Green, T., Saal, D., Marie, H., Neve, R., Nestler, E.J., and Malenka, R.C. (2006) CREB modulates excitability of nucleus accumbens neurons. *Nat. Neurosci*. **9,** 475–477.
6. Carlezon, W.A., Jr., Duman, R.S., and Nestler, E.J. (2005) The many faces of CREB. *Trends Neurosci*. **28,** 436–445.
7. Robinson, T.E. and Kolb, B. (2004) Structural plasticity associated with exposure to drugs of abuse. *Neuropharmacology* **47(Suppl 1),** 33–46.
8. Carpenter-Hyland, E.P. and Chandler, L.J. (2006) Homeostatic plasticity during alcohol exposure promotes enlargement of dendritic spines. *Eur. J. Neurosci.*, **24,** 3496–3506.
9. Carpenter-Hyland, E.P. and Chandler, L.J. (2007) Adaptive plasticity of NMDA receptors and dendritic spines: implications for enhanced vulnerability of the adolescent brain to alcohol addiction. *Pharmacol Biochem Behav*. **86,** 200–208.
10. Adams, C.E. and Stevens, K.E. (2007) Evidence for a role of nicotinic acetylcholine receptors in schizophrenia. *Front. Biosci*. **12**, 4755–4772.
11. Berman, J.A., Talmage, D.A., and Role L.W. (2007) Cholinergic circuits and signaling in the pathophysiology of schizophrenia. *Int. Rev. Neurobiol*. **78,** 193–223.
12. Everitt, B.J. and Wolf, M.E. (2002) Psychomotor stimulant addiction: a neural systems perspective. *J. Neurosci.* **22**, 3312–3320.
13. Kalivas, P.W. and Volkow, N.D. (2005) The neural basis of addiction: a pathology of motivation and choice. *Am. J. Psychiatry* **162**, 1403–1413.
14. Kalivas P.W. and Hu, X.T. (2006) Exciting inhibition in psychostimulant addiction. *Trends Neurosci.* **29**, 610–616.
15. Kourrich, S., Rothwell, P.E., Klug, J.R., and Thomas, M.J. (2007) Cocaine experience controls bidirectional synaptic plasticity in the nucleus accumbens. *J. Neurosci*. **27**, 7921–7928.
16. Boudreau, A.C. and Wolf, M.E. (2005) Behavioral sensitization to cocaine is associated with increased AMPA receptor surface expression in the nucleus accumbens. *J. Neurosci*. **25**, 9144–9151.
17. Boudreau, A.C., Reimers, J.M., Milovanovic, M., and Wolf, M.E. (In press) Cell surface AMPA receptors in the rat nucleus accumbens increase during cocaine withdrawal but internalize upon cocaine challenge in association with altered activation of mitogen-activated protein kinases. *J. Neurosci.*

18. Martin, M., Chen, B.T., Hopf, F.W., Bowers, M.S., and Bonci, A. (2006) Cocaine self-administration selectively abolishes LTD in the core of the nucleus accumbens. *Nat. Neurosci*. **9**, 868–869.
19. Hu, X.T., Basu, S., and White, F.J. (2004) Repeated cocaine administration suppresses HVA-$Ca2^+$ potentials and enhances activity of $K^+$ channels in rat nucleus accumbens neurons. *J. Neurophysiol*. **92,** 1597–1607.
20. Peoples, L.L. and Cavanaugh, D.J. (2003). Differential changes in signal and background firing of accumbal neurons during cocaine self-administration. *J. Neurophysiol*. **90**, 993–1010.
21. Peoples, L.L., Kravitz, A.V., Lynch, K.G., and Cavanaugh, D.J. (2007) Accumbal neurons that are activated during cocaine self-administration are spared from inhibitory effects of repeated cocaine self-administration. *Neuropsychopharmacology* **32**, 1141–1158.
22. Ghitza, U.E., Fabbricatore, A.T., Prokopenko, V., Pawlak, A.P., and West, M.O. (2003) Persistent cue-evoked activity of accumbens neurons after prolonged abstinence from self-administered cocaine. *J. Neurosci*. **23**, 7239–7245.
23. Hollander, J.A. and Carelli, R.M. (2005) Abstinence from cocaine self-administration heightens neural encoding of goal-directed behaviors in the accumbens. *Neuropsychopharmacology* **30**, 1464–1474.
24. Hollander, J.A. and Carelli, R.M. (2007) Cocaine-associated stimuli increase cocaine seeking and activate accumbens core neurons after abstinence. *J. Neurosci*. **27**, 3535–3539.
25. Durstewitz, D. and Seamans, J.K. (2002) The computational role of dopamine D1 receptors in working memory. *Neural Netw*. **15**, 561–572.
26. McClure, S.M., Daw, N.D., and Montague, P.R. (2003) A computational substrate for incentive salience. *Trends Neurosci*. **26**, 423–428.
27. Redish, A.D. (2004) Addiction as a computational process gone awry. *Science* **306**, 1944–1947.
28. Seamans, J.K. and Yang, C.R. (2004) The principal features and mechanisms of dopamine modulation in the prefrontal cortex. *Prog. Neurobiol*. **74**, 1–57.
29. Komendantov, A.O., Komendantova, O.G., Johnson, S.W. and Canavier, C.C. (2004) A modeling study suggests complementary roles for $GABA_A$ and NMDA receptors and the SK channel in regulating the firing pattern in midbrain dopamine neurons. *J. Neurophysiol*. **91**, 346–357.
30. Wolf, J.A., Moyer, J.T., Lazarewicz, M.T., Contreras, D., Benoit-Marand, M., O'Donnell, P., and Finkel, L.H. (2005) NMDA/AMPA ratio impacts state transitions and entrainment to oscillations in a computational model of the nucleus accumbens medium spiny projection neuron. *J. Neurosci*. **25**, 9080–9095.

**This article should be cited as follows:**

Volman, S.F. (2007) Evaluating the functional importance of neuroadaptions in addiction. *TheScientificWorldJOURNAL* **7(S2)**, 4–8. DOI 10.1100/tsw.2007.226.
